# My Deep Sea, My Backyard: a pilot study to build capacity for global deep-ocean exploration and research

**DOI:** 10.1098/rstb.2021.0121

**Published:** 2022-07-04

**Authors:** Diva J. Amon, Randi D. Rotjan, Brian R. C. Kennedy, Gerard Alleng, Rafael Anta, Eriatera Aram, Thera Edwards, Marcia Creary-Ford, Kristina M. Gjerde, Judith Gobin, Laura-Ashley Henderson, Alexis Hope, Raquel Khan Ali, Sebastian Lanser, Keith Lewis, Hannah Lochan, Scott MacLean, Nabuti Mwemwenikarawa, Brennan Phillips, Betarim Rimon, Stacey-Ann Sarjursingh, Tooreka Teemari, Aranteiti Tekiau, Alan Turchik, Henri Vallès, Kareati Waysang, Katherine L. C. Bell

**Affiliations:** ^1^ SpeSeas, D'Abadie, Trinidad and Tobago; ^2^ Natural History Museum, London SW5 7BD, UK; ^3^ Department of Biology, Boston University, Boston, MA 02115, USA; ^4^ Inter-American Development Bank, Washington, DC, USA; ^5^ Coastal Fisheries Division, Ministry of Fisheries & Marine Resources Development, Bairiki, Kiribati; ^6^ Department of Geography and Geology, The University of the West Indies-Centre for Marine Sciences, Mona Campus, Kingston, Jamaica; ^7^ The University of the West Indies-Centre for Marine Sciences, Mona Campus, Kingston, Jamaica; ^8^ IUCN Global Marine and Polar Programme and World Commission on Protected Areas, Cambridge, MA 02 02138, USA; ^9^ The University of the West Indies, St Augustine Campus, Saint Augustine, Trinidad and Tobago; ^10^ MIT Media Lab, Cambridge, MA 02139, USA; ^11^ COAST Foundation, Chaguaramas, Trinidad and Tobago; ^12^ Phoenix Islands Protected Area Conservation Trust, Tarawa, Kiribati; ^13^ Department of Ocean Engineering and Graduate School of Oceanography, University of Rhode Island, Narragansett, RI 02882, USA; ^14^ Independent Consultant, Tarawa, Kiribati; ^15^ National Institute of Higher Education, Research, Science and Technology, Port of Spain, Trinidad and Tobago; ^16^ Exploration Technology Lab, National Geographic Society, Washington, DC, USA; ^17^ The University of the West Indies, Cave Hill Campus, Cave Hill, Barbados; ^18^ Phoenix Islands Protected Area Implementation Office, Tarawa, Kiribati; ^19^ Ocean Discovery League, Saunderstown, RI 02874, USA

**Keywords:** capacity building, science, exploration, small island developing state, Kiribati, Trinidad and Tobago

## Abstract

The deep ocean is the largest ecosystem on the planet, constituting greater than 90% of all habitable space. Over three-quarters of countries globally have deep ocean within their Exclusive Economic Zones. While maintaining deep-ocean function is key to ensuring planetary health, deficiencies in knowledge and governance, as well as inequitable global capacity, challenge our ability to safeguard the resilience of this vast realm, leaving the fate of the deep ocean in the hands of a few. Historically, deep-ocean scientific exploration and research have been the purview of a limited number of nations, resulting in most of humankind not knowing the deep ocean within their national jurisdiction or beyond. In this article, we highlight the inequities and need for increased deep-ocean knowledge generation, and discuss experiences in piloting an innovative project ‘My Deep Sea, My Backyard’ toward this goal. Recognizing that many deep-ocean endeavours take place in countries without deep-ocean access, this project aimed to reduce dependency on external expertise and promote local efforts in two small island developing states, Trinidad and Tobago and Kiribati, to explore their deep-sea backyards using comparatively low-cost technology while building lasting in-country capacity. We share lessons learned so future efforts can bring us closer to achieving this goal.

This article is part of the theme issue ‘Nurturing resilient marine ecosystems’.

## 1. Introduction

Humankind is at a critical juncture for stewarding Earth's ocean. Negotiations are close to heralding a commitment toward protecting 30% of the ocean by 2030 at the Conference of the Parties to the Convention on Biological Diversity (CBD) in 2022, and the UN Decade of Ocean Science for Sustainable Development has just begun [1]. At the same time, global attention is increasingly turning to the promise of the Blue Economy, with largely unrestrained expansion of human activities and associated impacts into all areas of the ocean [2–4]. This is despite the United Nations Environment Programme Finance Initiative [5, p. 16] defining a truly sustainable ocean economy as one that will provide ‘social and economic benefits for current and future generations; restore, protect and maintain diverse, productive and resilient ecosystems; and is based on clean technologies, renewable energy and circular material flows'. It is clear that all of these needs can only be met with a sustained healthy and resilient ocean, which critically and urgently requires scientifically informed, holistic decision-making and management [6].

Given that the deep ocean (greater than 200 m) is the largest ecosystem on the planet, constituting 90% of all habitable space [7], it follows that maintaining its function is key to ensuring planetary health. The deep ocean plays a key role in providing provisioning (e.g. fisheries supporting diets and livelihoods), regulating (e.g. climate regulation via carbon sequestration and storage) and cultural services (e.g. spiritual significance) [8,9]. However, with inadequate stewardship owing to deficiencies in knowledge and governance, as well as inequitable global capacity (i.e. the mix of human resource, technology, infrastructure, mechanisms, finance, access and other more nuanced types) [10], the fate of the deep ocean continues to remain in the hands of just a few, even though most of the world has a vested interest in deep-ocean ecosystems and issues [11].

**Figure 1.**
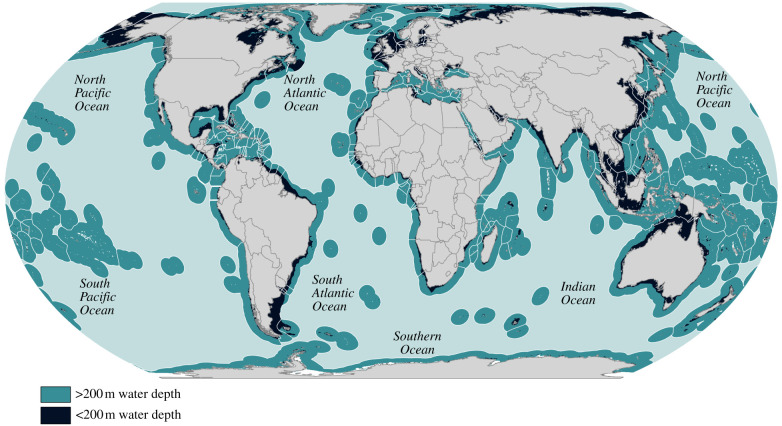
The global distribution of deep ocean by country. EEZ data from the Maritime Boundaries Geodatabase (Flanders Marine Institute, 2019), 200 m contour shape file provided by Natural Earth (https://www.naturalearthdata.com/downloads/10m-physical-vectors/10m-bathymetry/).

The deep ocean is not just ‘out there’; rather, it comprises a significant percentage of national waters and seabed. For example, about three-quarters (73%) of geographical areas globally (169 out of 231) have deep ocean within their Exclusive Economic Zones (EEZs) (figure 1 and table 1). Here, we define ‘geographical areas’ as sovereign nations and territories that are classified by the UN M49 standard as ‘developed', ‘developing’ or ‘small island developing states (SIDS)’ (https://unstats.un.org/unsd/methodology/m49/#geo-regions). Territories that are not included in the M49 classification are included with their sovereign state. By region, the largest number of geographical areas with deep-ocean areas are in Latin America and the Caribbean (43 geographical areas), while the largest deep-ocean area within national jurisdiction is within Oceania (total 36 366 000 km^2^) (figure 1 and table 1). Of the geographical areas with deep ocean, 62% have developing economies. Furthermore, 61.9% of geographical areas globally have deep ocean occupying more than 50% of their national jurisdictions (143 out of 231), and for nearly one-third of geographical areas (32.5%) the deep ocean occupies 90% or more of their national jurisdictions (75 out of 231) (figure 1). This includes 81.6% of all SIDS (40 out of 49) (figure 1).

**Table 1.** Global summary of areas with deep ocean within national jurisdictions. ‘Geographical areas’ are defined as sovereign nations and territories with economies identified as developed, developing or small island developing states according to the UN M49 standard. Territories that do not have UN-classified economies are included with their sovereign nation. Data sources: (a) https://unstats.un.org/unsd/methodology/m49/#geo-regions; (b) *Sea Around Us concepts, design and data* (https://www.seaaroundus.org) [12].

	category	total deep-ocean area within national jurisdictions (km^2^)	% deep-ocean area of total deep ocean within national jurisdictions	total number of areas with deep ocean	% of total areas with deep ocean
all geographical areas with deep ocean	—	115 928 000	100	169	100
UN economy classification	developed economies	44 463 000	38	39	23
	developing economies	37 953 000	33	81	48
	small island developing states	33 512 000	29	49	29
geographical region	Africa	12 823 000	11	41	24
	Americas: Northern	14 007 000	12	5	3
	Americas: Latin & Caribbean	18 149 000	16	43	25
	Asia	17 920 000	15	29	17
	Europe	16 664 000	14	25	15
	Oceania	36 366 000	31	26	15

**Figure 2.**
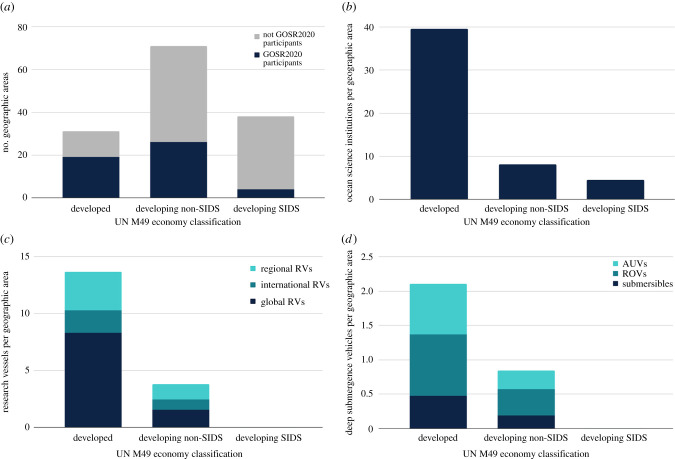
The distribution of ocean research capacity in geographical area with deep ocean. ‘Geographic areas’ are defined as sovereign nations and territories with economies identified as developed, developing, or small island developing states according to the UN M49 standard. Territories that do not have UN-classified economies are included with their sovereign nation. (*a*) Fifty-two geographical areas contributed to the Global Ocean Science Report 2020; of those, 47, both developing and developed, with deep ocean were surveyed. Geographical areas with economies in transition were not included in this figure as there were not enough data. (*b*) The average number of ocean science institutions per geographical area. (*c*) The average number of global, international and regional class research vessels (RVs) per geographical area. Local coastal (10–35 m in length) or smaller than 10 m vessels were not included as deep-ocean research typically requires larger vessels. (*d*) The average number of deep-submergence vehicles (submersibles, ROVs and/or AUVs) per geographical area. Data from the Global Ocean Science Report [11] and https://unstats.un.org/unsd/methodology/m49/#geo-regions.

For too long, deep-ocean scientific exploration and research has been the purview of a limited number of nations, entities and people. The grave mismatch between the percentage of deep-ocean area per geographical area and national access to the deep ocean for scientific study prevents most of humankind from knowing the deep ocean within their national jurisdictions or that of the global ocean commons (figure 1 and table 1). The most recent version of the Global Ocean Science Report (GOSR) [11] surveyed 49 geographical areas (as defined above) with deep ocean, of which developing geographical areas were underrepresented, and showed significant disparities in national capacities to undertake open-ocean and deep-ocean research. For example, there are an average of 40 ocean science institutions per developed geographical area that took part in the survey, while non-SIDS developing geographical areas had only eight institutions per geographical area, and developing SIDS had 4.5 institutions per geographical area that participated in the survey (figure 2). According to the GOSR, developed geographical areas, therefore, have 5–10 times more institutions in existence or actively engaged, compared with developing geographical areas. On average, developed geographical areas also operate approximately 14 global, international and/or regional class research vessels per geographical area (approx. 72% of the global fleet), while non-SIDS developing geographical areas operate approximately four vessels per geographical area, and SIDS operate zero (figure 2) (echoed by [13]). Furthermore, the five geographical areas with the most research vessels (USA, Canada, Japan, Norway and Australia, respectively) account for 51% of the research vessels greater than 35 m in length reported globally. On average, approximately 2.5 times more developed geographical areas have deep submergence vehicles (submersibles, remotely operated vehicles (ROVs), and autonomous underwater vehicles (AUVs)) than developing geographical areas, and SIDS operate none. It is important to note, however, that while GOSR includes the best data currently available, it still underrepresents the true disparity (e.g. France owns and operates scientific ROVs but it is not included in the GOSR data) (figure 2). As such, the disparity of assets is skewed, as is the capacity to use these assets. The first step to mitigating these disparities is to recognize that the global community needs to go beyond the existing data in the GOSR to truly understand the capacity for deep-sea exploration and research for every country with deep ocean within their national jurisdiction. To that end, the Global Deep-Sea Capacity Assessment is currently underway as a UN Ocean Decade Activity (https://deepseacapacity.pubpub.org/).

There are growing calls for a paradigm shift in exploration and science that includes increased access, inclusion and equity, including with regard to the deep ocean, especially given the rapidly advancing technology and imminent Blue Economy applications (e.g. [1,14,15]). However, while the goals for such paradigm shifts are laudable, programmes to address these issues are often difficult to implement, unidirectional in nature, challenging to quantify and/or can ultimately perpetuate the fundamental inequities that they are trying to address [10,16,17].

To meet the need for increased deep-sea capacity, we conducted a small pilot project, ‘My Deep Sea, My Backyard’ (MDSMBY). Recognizing that many deep-ocean endeavours take place in countries with deep ocean, but without deep-ocean access, MDSMBY aimed to grow deep-ocean capacity in two countries, Trinidad and Tobago, and Kiribati, to explore their deep-sea backyards using comparatively low-cost technology while building lasting in-country capacity. These pilot projects offered a glimpse into a series of issues not yet fully addressed by the ocean community. In an effort to accelerate progress in deep-ocean access, capacity and equity, here we share our experiences, including the missteps, challenges, successes and lessons learned, so that future efforts will bring us collectively closer to achieving a more equitable ocean.

## 2. My Deep Sea, My Backyard

MDSMBY was spawned during ‘Here Be Dragons’, a convening of explorers, innovators, artists, scientists and storytellers to identify the uncharted territories that still exist in ocean exploration and storytelling, and address gaps in our understanding and sharing of the ocean, hosted by K. Bell at MIT Media Lab in February 2018. From her own experience in Trinidad and Tobago, D. Amon questioned how deep-ocean exploration could be enabled in countries without the means currently. At the same time, R. Rotjan, in her role as Co-Chief Scientist of the Phoenix Islands Protected Area Conservation Trust, was facing similar challenges enabling deep-sea capacity in Kiribati. This led to a self-selected collaborative team coming together to conceptualize and propose potential solutions.

With support from The National Geographic Society ($50 000 US) and the Inter-American Development Bank (IDB) ($15 000 US), a pilot study was designed to provide deep-ocean access and increased technological capacity in Kiribati and Trinidad and Tobago. These two SIDS were chosen because of pre-existing relationships between collaborators and/or a need expressed by nationals. The approach had four goals:

1. To build lasting capacity evidenced by country nationals (ideally a scientist, student and communicator) exploring the ocean and then communicating the findings;
2. To enable technology transfer via access to new, innovative, (comparatively) low-cost deep-ocean technology that can be used from any platform;
3. To engage a broad group of stakeholders in deep-sea exploration and science;
4. To collaborate equitably while working within the cultural norms and customs of each country and according to the needs, constraints or interests of in-country partners.

## 3. Exploring the deep ocean in Kiribati

The Republic of Kiribati is a least developed country (LDC) and SIDS, with a lower-middle-income economy by *per capita* Gross National Income (GNI), located in Oceania. Kiribati consists of 33 islands spanning 46° of longitude across three archipelagos stretched across all four hemispheres, in the equatorial Pacific Ocean. These archipelagos constitute the 12th largest country by ocean area but the 24th smallest country by land area. Their average elevation is only 3 m above sea level, but the maximum ocean depth is greater than 7000 m. The island of Tarawa holds most of the residential population (approx. 64 000 people of a total 117 000) and is the country's capital. Tarawa is a mid-ocean atoll that contains a large shallow lagoon and is surrounded by a fringing reef that rapidly slopes into the depths. Deep water (greater than 200 m) can be found less than 4–5 km from any point on the land and occupies 99.6% of the EEZ.

Kiribati has a long history of industrial offshore purse-seine, longline and pole-and-line tuna fishing by both foreign-flagged and locally flagged vessels. Although these activities have been concentrated in the surface layer of the ocean, many of these commercially important species and those they rely on partially dwell in the deep ocean. Additionally, Kiribati sponsors a deep-seabed mining exploration licence in the Clarion–Clipperton Zone (CCZ), granted by the International Seabed Authority (ISA), for Marawa Research and Exploration Ltd [18]. Kiribati is also home to the Phoenix Islands Protected Area (PIPA), the largest and deepest UNESCO World Heritage Site, which has been a focal point for deep-ocean exploration in recent years [19–21]. As such, the need for increased deep-ocean literacy and capacity in Kiribati is timely and related to both industry and conservation initiatives. However, this is currently not possible as Kiribati does not have any deep-ocean scientific experts, deep-ocean scientific technology or national research vessels capable of venturing offshore to access the deep ocean.

The MDSMBY Kiribati case study was jointly undertaken by the Republic of Kiribati, Boston University, the University of Rhode Island, the PIPA Conservation Trust and the PIPA Implementation Office. However, the project was co-led by the PIPA Co-Chief Scientists, the Executive Director of the PIPA Conservation Trust and the PIPA Education and Outreach Officer in the PIPA Implementation Office. Consultations among these parties occurred prior to bringing technology to Tarawa to discuss the concept and basic logistics. Once in-country, several stakeholder meetings and training sessions were held to introduce deep-ocean ecosystems, demonstrate the technology and demonstrate analysis pathways. Decisions on where to deploy technology were made in real-time by the participants but were partly influenced by vessel availability, fuel resources, weather and participant interest in sites and locations. It became clear that, because this was the first-ever exploration of the deep ocean in the Gilbert Archipelago, the initial location was less important than the training and proof-of-concept demonstration with the technology. Transferring training and technology was the priority so that the Kiribati could then explore their waters, implementing their own study designs to meet their interests and objectives.

**Figure 3.**
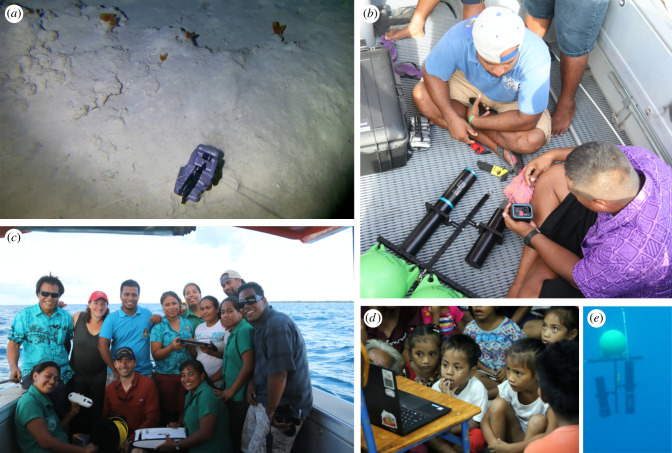
‘My Deep Sea, My Backyard’ in Kiribati. (*a*) Tarawa seafloor image captured by the ReelCam from approximately 800 m, with benthic ctenophores and soft sediment. (*b*) Government of Kiribati fisheries staff prepared the ReelCam for deployment. (*c*) The 2018 project team. (*d*) Kiribati school children see the deep ocean for the first time. (*e*) Deep-ocean ReelCam descending from the surface.

The MDSMBY Kiribati case study used innovative deep-ocean technology, the ‘ReelCam’, which was a custom-built camera system developed at the University of Rhode Island (B. Phillips) and deployed on a deep-ocean electric fishing reel [22] (figure 3). It consisted of a GroupB pressure housing for a GoPro type HD camera, and an LED light (3500 lm) mounted on an aluminium frame with two commercial spherical trawl floats mounted on the top of the Euro Products frame. Approximately 10 kg of dive weights were hung approximately 1 m below the camera via a monofilament fishing line. The entire camera and weight assembly were attached to a Lindgren-Pitman electronic fishing reel loaded with a braided Tuf-Line fishing line. The camera package and weights were designed to be negatively buoyant and sink until the weights hit the seafloor. At the same time, the camera with its floats was positively buoyant and designed to hang suspended approximately a metre above the seafloor, thereby allowing a well-lit view of the benthos. Star-Oddi depth and temperature logger sensors added the potential for basic oceanographic metadata. This system provided relatively easy-to-assemble deep-submergence exploration tools, constructed from commercially available products with an entire cost approximating $10 000 US. OpenROV's Trident ROVs were also used as a shallow-water and mesophotic exploration tool, chosen for ease of use and freedom of platform (can be deployed from any platform).

The team conducted over a week of training (25 June to 2 July 2018), both in the classroom and in the field, for government fisheries officers, University of the South Pacific (USP) students, science communicators and outreach officers and multiple representatives from related government agencies (Ministry of Environment, Land and Agricultural Development (MELAD), Tourism, Fisheries). Training on use of the ocean-exploration technology was undertaken onshore and in the field (figure 3). There was also a stakeholder consultation for individuals representing several arms of the Government of Kiribati (e.g. MELAD, Fisheries, Tourism, etc.), the police, several local villages, and USP Tarawa Campus. Stakeholder conversations were held to initiate discussions about the technology, the deep-ocean ecosystem, and how this project could help enable stakeholder goals. There was no prescribed question or hypothesis driving the consultation. Instead, open-ended stakeholder consultation facilitated two-way conversation and collaboration.

During training, the first successful deployments of the ReelCam were undertaken in true collaboration with in-country partners (figure 3). The ReelCam successfully captured the first deep-ocean images in the Gilbert Archipelago between North and South Tarawa. These images showed benthic ctenophores at approximately 800 m depth, and were a proof of concept for the technology, platform and approach. The rationale for deployment was mainly to enable technology and training, and to demonstrate proof of concept. Once technology was transferred and training complete, stakeholders in-country could deploy the ReelCam or the Trident ROVs to achieve their own goals and interests. The Trident ROVs were used by the Ministry of Fisheries to conduct shallow coral surveys and exploration, and as a communications tool. Real-time viewing on a handheld tablet provided instant excitement and engagement, which was a powerful tool for participants. The Trident ROV's ease of use, which included deployment from many platforms such as bridges, causeways, small pangas (outboard fishing vessels) and repurposed public-sector boats such as police vessels, was a large contributor to its success. In particular, the Trident ROVs were an effective tool for helping participants to become comfortable with ROVs and higher-tech equipment and helping to visualize familiar (shallow) and novel (mesophotic) environments with the 100 m tether. To our knowledge, the mesophotic images and video captured by the Trident ROV were the first mesophotic data from the Gilbert Archipelago.

The opportunity to demonstrate and use these deep-ocean tools was key to facilitating communication about the deep ocean and the pilot study, as well as the identification of priority areas for investigation, with communities, villages, schools and the government in Kiribati. Outreach efforts included engagements with more than 2500 students from elementary through college-school level, as well as meetings with village elders, fishers and the highest levels of government (figure 3). Additionally, this project gained coverage in multiple online blogs and articles, and a national radio interview.

There were, however, also challenges during the Kiribati case study, namely with technology. The ReelCam system lacked real-time feedback while submerged, making it difficult to determine when the system had reached the seafloor given there are strong currents around Kiribati and none of the available vessels had a sufficient depth-finder. Unfortunately, despite the clear interest in conducting this work by partners and workshop participants, the ReelCam was not used at all in the year following training, primarily because there was no funding for fuel for a vessel to go into deep water. The Trident ROV also had challenges in Kiribati, including difficulty facilitating software updates because of the limited internet. Additionally, the Trident ROV would be more successful as a science tool with additional sensors to enable measurements of depth and other oceanographic parameters such as temperature, O_2_ and salinity. Finally, the ReelCam instrumentation was lost at sea during the second training period, and COVID-19 impeded delivery of a replacement. However, interest in the technology and its use in Kiribati has been consistently communicated, which is an indication of the pilot project's success.

## 4. Exploring the deep ocean in Trinidad and Tobago

Trinidad and Tobago is a developing country, high-income economy by *per capita* GNI, and a SIDS. It is the most southerly island nation of the Caribbean archipelago and borders the South American mainland. The island of Trinidad holds most of the residential population (1.3 million people of a total 1.4 million) as well as the country's capital, Port of Spain. Trinidad and Tobago sits on an extensive continental shelf that stretches 80–100 km from Trinidad's east coast, a much greater distance than those observed within the EEZs of other Caribbean nations. Despite this, Trinidad and Tobago's deep ocean still occupies over 54 000 km^2^ (69.1%) of the EEZ, with depths ranging between 200 and 3500 m.

Trinidad and Tobago has large reserves of oil and natural gas, which has resulted in a prolific industry that has sustained the economy for decades and is now extending into deeper offshore areas, including those known to harbour deep-ocean methane seeps and corals within the EEZ [23,24]. A small semi-industrial longline fishing fleet that targets highly migratory pelagic species operates in the deep ocean also (http://www.fao.org/fishery/facp/TTO/en). The Government of the Republic of Trinidad and Tobago has also developed a National Protected Areas Systems Plan, which includes five areas that encompass deep ocean [25]. As such, the need for increased deep-ocean literacy and capacity in Trinidad and Tobago is timely and related to both industry and conservation initiatives. This is currently not possible as Trinidad and Tobago does not have deep-ocean scientific technology or any national research vessels capable of venturing offshore to access the deep ocean; however there are in-country deep-ocean scientific experts.

The MDSMBY Trinidad and Tobago case study was jointly undertaken by the COAST Foundation, Inter-American Development Bank (IADB), MIT Media Lab, National Geographic Society (NGS), the National Institute of Higher Education, Research, Science and Technology (NIHERST), SpeSeas and The University of the West Indies, St Augustine. Local scientist D. Amon led the Trinidad and Tobago case study. The team conducted stakeholder consultations at The University of the West Indies with over 80 individuals representing several sections of the Government of the Republic of Trinidad and Tobago (e.g. Ministry of Planning and Development, Environmental Management Authority), the Coast Guard, the oil and gas industry, local environmental non-governmental organizations (NGOs), the National Institute of Higher Education Research, Science and Technology, and other academic institutions (e.g. The University of the West Indies, University of Trinidad and Tobago). This opportunity facilitated two-way conversation and collaboration about the deep-ocean ecosystem (including the scale and relevance of it in the Caribbean, as well as fundamental characteristics of the environment and biology); the pilot project including the technology, key societal drivers for exploration and how this project could help enable stakeholder goals. From this, the key question was asked ‘Where in Trinidad and Tobago's deep-sea backyard is of highest priority to explore and characterize?’ which led to the identification of priority reasons for and areas of investigation (on maps of the EEZ) during the stakeholder consultation. These reasons included, for instance, understanding areas before deep-ocean oil and gas exploitation, exploring interesting bathymetric features to ‘see what lives there’, searching for shipwrecks, and using this exploration ‘to get people excited about the ocean’ and ‘demystify the ocean and make it more accessible in a relatively safe way’.

**Figure 4.**
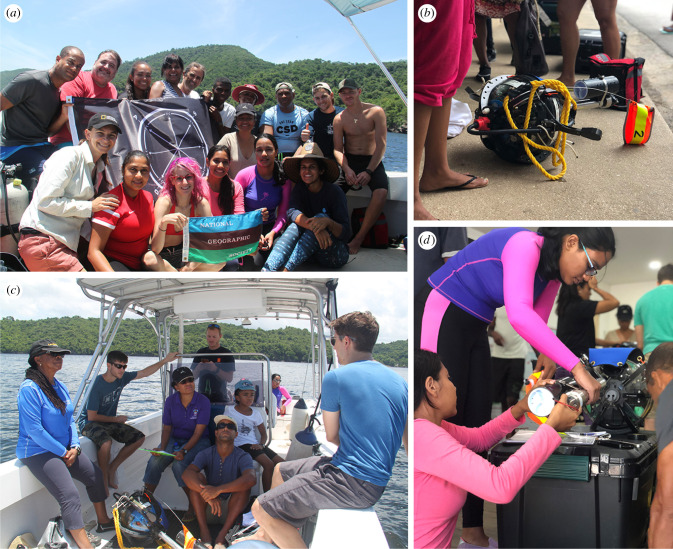
‘My Deep Sea, My Backyard’ in Trinidad and Tobago. (*a*) The project team. (*b*) The National Geographic Deep-Sea Drop Camera. (*c*) Participants learning to deploy the National Geographic Deep-Sea Drop Camera at sea. (*d*) Participants learning to prepare the National Geographic Deep-Sea Drop Camera for deployment.

The MDSMBY Trinidad and Tobago case study used an innovative Deep-Sea Drop Camera developed by National Geographic's Exploration Technology Lab (ExTech) (figure 4). This is an untethered free-falling system capable of diving to 6000 m and staying submerged for more than a day [26]. An OpenROV Trident ROV and a Blue Robotics BlueROV2 ROV, both rated to 100 m depth, were also used as shallow-water and mesophotic exploration tools for their ease of use and freedom of platform.

One week of training (13–17 August 2018) was conducted with two scientists, three students, a science communicator and four marine engineers from Trinidad and Tobago. Additionally, two scientists participated from The University of the West Indies Mona Campus in Jamaica and one scientist from The University of the West Indies Cave Hill Campus in Barbados. Training on use of the ocean-exploration technology was undertaken onshore and in the field (figure 4). Unfortunately, the training was impeded by the airline temporarily losing the Deep-Sea Drop Camera, leading to its late arrival, preventing the Trinidad and Tobago team from getting enough hands-on time with the equipment to feel fully comfortable during the training workshop. To counteract the lost hands-on training time during the training week, one of the students joined project partners during another expedition in Bermuda after the initial training period.

There were many challenges during the Trinidad and Tobago case study. There were several attempts during the year following training to undertake deployments with the Deep-Sea Drop Camera. Unfortunately, recurring technical issues with the Deep-Sea Drop Camera, which could not be resolved without the in-person assistance of an ExTech engineer, led to long periods of inactive use. This was further complicated by the need to mobilize ad hoc teams quickly given the reliance on opportunistic vessels within budget range capable of transiting the long distances to Trinidad and Tobago's deep ocean, especially as the government of Trinidad and Tobago does not have such research vessels. Although suitable vessels were sourced, vessel availability usually aligned with periods when the equipment was not working or key members of the Trinidad and Tobago team were abroad, preventing research trips from moving forward. Despite the clear interest in conducting this work, there were no successful Deep-Sea Drop Camera deployments during the project.

While the training and exploration goals fell short of what was co-developed, the Trinidad and Tobago team engaged over 250 students from elementary through high-school level about the deep ocean, its inhabitants, and its exploration. The Trident ROV was not used for science but was used to assist SpeSeas as a communication tool for participants to understand the power of this type of technology and exploration for marine science and stewardship. In particular, the Trident ROV was effective for helping students and other participants to become comfortable with ROVs, higher-tech equipment, and remote ocean exploration. Real-time viewing provided instant gratification, excitement and engagement. Additionally, there was island-wide coverage on national television, radio and newspapers, as well as on multiple online blogs and articles.

## 5. Lessons learned

There have been many recently published insights on the steps needed to achieve genuinely inclusive and equitable ocean scientific research and conservation, including capacity-building initiatives [10,14–17,27–30]. In the interest of propelling the ocean-science community at large to critically assess past capacity-building projects and improve others in the present and future, we have weighed the MDSMBY pilot process and outcomes against our initial goals (reducing dependency on external expertise and promoting local research efforts in Kiribati and Trinidad and Tobago). Details on all components are articulated below and in table 2.

**Table 2.** A critical assessment of ‘My Deep Sea, My Backyard’ (MDSMBY) in Kiribati and Trinidad and Tobago.

MDSMBY goals	Kiribati process and outcomes	Trinidad and Tobago process and outcomes
collaborate equitably while working within the cultural norms and customs of each country and according to the needs, constraints, or interests of in-country partners	successes: – had several i-Kiribati partners – worked within the maneaba system, the heart of Kiribati community – partnered several local and international organizations to co-develop outputs and execute the project	successes: – co-led by a Trinidad and Tobago national – partnered with several local and international organizations to co-develop outputs and execute the project – a stakeholder consultation was undertaken to identify the needs, constraints and interests of the national community
	challenges: – there was not adequate funding/compensation for most individuals/organizations that were executing the project, both in-country and foreign	challenges: – there was not adequate funding/compensation for most individuals/organizations that were executing the project, both in-country and foreign
build lasting capacity evidenced by country nationals (ideally a scientist, student and communicator) exploring the ocean and then communicating the findings	successes: – conducted classroom and in-field training – workshop participants ranged from early to senior career staff from the fisheries and environment ministries who are in positions to shape policy – Trident ROV continued to be used for scientific surveys – communication of findings was done bilingually, by and for country nationals both during the project and beyond – findings from this (and other) projects have become part of the overall outreach effort for the PIPA Implementation Office	successes: – conducted classroom and in-field training – workshop participants included early and senior career researchers from Trinidad and Tobago, as well as Jamaica and Barbados – Trident ROV continued to be used for outreach
	challenges: – long-term support, financial, technical, educational, or otherwise, was not provided as this was only a pilot study – timeline chosen was unrealistic – metrics were not chosen to monitor and ensure effectiveness of capacity building – the loss of half of the ReelCam at sea has prohibited further use – not enough data were collected to enable training on species identification and data analysis, or to contextualize the implications of discoveries	challenges: – long-term support, financial, technical, educational, or otherwise, was not provided as this was only a pilot study – timeline chosen was unrealistic – metrics were not chosen to monitor and ensure effectiveness of capacity building – not enough field training was provided – in Trinidad, deep ocean is >100 km from the major port, requiring specific vessels that were not easily available – no deep-ocean data were collected to enable training on species identification and data analysis, or to contextualize the implications of discoveries
enable technology transfer via access to new, innovative, (comparatively) low-cost deep-ocean technology that can be used from any platform	successes: – technology was comparatively low-cost (approx. $10 000 US) – technology was low-logistic and easy to use, enabling deployment from vessels available in-country – technology successfully captured mesophotic (Trident ROV) and deep-ocean (ReelCam) imagery in Tarawa, thus completing the first-ever deep-ocean explorations on that island and within the Gilbert Archipelago – technology was successfully used by country nationals from beginning to end, multiple times, thus building comfort and familiarity – technology will remain in-country permanently, enabling future use	successes: – technology was comparatively low-cost (approx. $15 000 US) – the technology was applicable to the country's deep-ocean environment
	challenges: – Trident ROV software upgrades are difficult to download, given the limited internet bandwidth in-country – sustained funding not provided for fuel for a vessel to access deep water – repairs and replacement parts cannot be sourced in-country, so loss or damage necessitates long wait-times for replacement or repair	challenges: – technology required repairs on several occasions, which could not be undertaken by in-country participants given complexity; this combined with other factors inhibited the successful use of the DropCam – repairs and replacement parts could not be sourced in-country, so loss or damage necessitated long wait-times for replacement or repair – technology was on loan; it did not remain in-country permanently, preventing use beyond the scope of the project – locating locally-available vessels was challenging given proximity to the deep ocean – not enough training in use of technology and working offshore was provided – use of the technology was not built into existing infrastructure or institutions
engage a broad group of stakeholders in deep-sea exploration and science	successes: – engaged with numerous stakeholder sectors, from government to elementary school children – the Trident ROV was a useful tool for outreach	successes: – engaged with numerous stakeholder sectors, from government to elementary school children – the Trident ROV was a useful tool for outreach
	challenges: – one-time presentations are not sufficient to fully engage a national-level conversation – pathway for more meaningful engagement (e.g. by interested students) does not yet exist – metrics that measure and assess the learning from engagement were not used	challenges: – a lack of results meant communication of findings did not occur – metrics that measure and assess the learning from engagement were not utilized

Ultimately, this pilot study consisted of many elements that could be used in a longer-term project; however, they will require tailored and co-designed amendments to achieve the goals and meet the needs of each specific country (tables 2 and 3). Successful elements of both the Kiribati and Trinidad and Tobago case studies included partnership and collaboration between several local, regional and international organizations to co-develop outputs, and execute and co-lead the projects. Both teams inherently worked within the culture of the country and spoke to country-specific priorities. For the latter, stakeholder consultations were also undertaken to identify the needs, constraints and interests of the national community. However, for longer-term endeavours, more in-depth discussion and co-development to identify training priorities as well as the necessary support to enable these should take place between all partners. Additionally, culturally appropriate metrics (beyond the number of participants) should be chosen to monitor and ensure effectiveness of capacity building [10].

**Table 3.** Recommendations for undertaking deep-ocean capacity-building projects.

leadership and management	– project should be led or co-led, from design via implementation to output, by a national – use strategic partners, locally and internationally, to provide technology, training, and/or advice, although not to the detriment of the in-country partners
respect and recognition	– work within cultural processes and norms of the community – understand local issues related to and priorities for deep-ocean exploration and research; there may be differences in the priorities of local versus foreign partners
technology	– technology should be low-cost, recognizing that low-cost may have a different meaning for developing versus developed countries – technology should be easy to use and repair without requiring in-person foreign intervention – technology should require easy logistics i.e. be deployable from locally available vessels – technology should be applicable to the country's deep-ocean environment, e.g. have capabilities to answer the desired questions – technology should remain in-country indefinitely
training	– training priorities should be co-developed with in-country partners – training priorities could include data collection, data processing and analysis, operations and logistics, expedition planning and execution, communications and storytelling – there is a need for formal metrics that measure and assess the long-term effectiveness of capacity-building measures in ocean science that goes beyond how many individuals were engaged
funding	– sustained funding, including potentially from a public source
outreach	– priorities for the engagement of stakeholders should be co-developed with in-country partners and commensurate with available resources, including with provisions for local languages – there is a need for formal metrics that measure and assess the learning due to outreach measures that goes beyond how many individuals were engaged
lasting capacity	– priorities for building lasting capacity should be co-developed with in-country partners – in line with long-term priority outcomes, metrics should be chosen to monitor and ensure effectiveness of capacity building – commitments should be secured for long-term, multi-year support, financial or otherwise – realistic timelines should be chosen, recognizing that building lasting capacity requires multiple years

Both case studies conducted classroom and field training, with some of the technology continuing to be used once training was complete. However, more active operationalization was hampered by inadequate financial support (e.g. funding/compensation) for most individuals and organizations that were executing the project, both in-country and abroad. Although this was designed to be a short-term pilot study, it was ultimately unrealistic to achieve the goals within a year.

The technology was comparatively low-cost. For context, this cost is relatively inexpensive compared with the majority of deep-ocean assets, which often cost millions of dollars to build and deploy. However, we recognize that $10 000 US may still be unaffordable for many. Ample time for training should also be included. In Kiribati, there was a revolving list of personnel during training, which allowed a wider group of people to be partially trained, but also created a challenge for reinforcing knowledge. If the technology required repair or replacement, this could not be undertaken without the intervention of foreign partners owing to lack of materials and supplies on-island. Also, the lost ReelCam has still not been replaced owing to travel restrictions associated with the COVID-19 pandemic, whereas in Trinidad and Tobago, repair was not possible without National Geographic ExTech engineers, partly inhibiting its successful use. However, the ReelCam is easily repaired if spare parts are available, which is a benefit to its use. In terms of longevity and enabling future use, both the ReelCam and multiple OpenROV Tridents were permanently gifted to Kiribati, whereas the DropCam was on loan to Trinidad and Tobago for only 1 year, hindering further work. Access to vessels capable of travelling approximately 100 km was challenging in Trinidad and Tobago, especially given equipment faults, personnel travel delays and equipment return deadlines, which provided a stark difference to Kiribati, where the deep ocean was much closer but vessels with adequate batteries to power the ReelCam were less common.

The example of vessel access stresses the importance of avoiding generalizations during planning, and instead coming up with tailored and co-designed solutions. Other areas for future improvement include ensuring that the use of given or loaned technology be built into existing infrastructure, institutions and jobs, so activities are easier to undertake and do not provide an added burden above and beyond existing obligations. In addition, participants did not receive any formal certificate or degree, so the utility of the training for career advancement is limited to the knowledge gained and the participation line on their curriculum vitae (CVs).

The outreach components in both countries were the most successful given the scale and breadth of activities. Through engagement with numerous stakeholder sectors, from government officials to elementary school children, priorities for the engagement of stakeholders were co-developed with in-country partners and in line with resources available. Additionally, generating awareness and interest in deep-ocean exploration, science and stewardship was certainly accomplished. In addition, these efforts have helped to inspire further deep-ocean work led by in-country individuals. Unfortunately, because the technology saw limited use in both countries, there were insufficient data generated. As such, communication of the findings was challenging or non-existent.

## 6. Recommendations

With our rapidly changing climate and increasing threats to the deep ocean, we need scientifically informed decisions, which require several orders of magnitude more scientific capacity to successfully undertake exploration and research globally. Here, we build on the experiences of MDSMBY within Kiribati and Trinidad and Tobago and outline recommendations for undertaking projects aimed at growing deep-ocean capacity (table 3). These recommendations span several categories, including leadership and management of the project, respect and recognition of cultural processes and priorities, technological challenges and advantages, training goals, outreach efforts, and specific ideas to build lasting capacity and to try to combat the disparities in deep-ocean capacities between countries and regions.

Tailored and co-designed multi-pronged approaches, rather than any single measure, are needed to successfully build lasting capacity, although these require more time, effort and funding [16]. Additionally, one-time activities are no substitute for long-term partnerships. Further, needs and goals must be within the remit of national priority and tied to value addition to the objectives. The timeline of most grants is incompatible with the long timescales needed to successfully build lasting capacity, necessitating sustained funding to achieve results, and thus collaboration with funding organizations is needed to reimagine the types and timescales of funding currently administered. Public funding can assist with facilitating this but will need to tailor support for each country based on the above-mentioned factors, the number of people needed to sufficiently build lasting capacity in-country, and the cost of personnel, fuel, ships, travel etc. per country.

For deep-ocean capacity-building projects to succeed, it is also clear that technology assets and training resources are necessary. However, equally necessary is the adequate provisioning of human and logistical resources. The history of volunteerism that is so common in developed-world environmental fields hampers participation of the already underrepresented. Providing reasonable salary support (in line with country norms) or embedding projects within existing workflow for full-time government employees, is necessary to catalyse and sustain local engagement. In addition, providing support for smaller resources, such as boat fuel, internet access and other incidentals, is also necessary and critical to mission success (detailed in tables 2 and 3), since in-country collaborators may not have the flexible resources needed to procure sustained logistic support.

Creating long-term project sustainability will likely be easier if the project can be embedded into an existing programme, project or entity. Tying the success of a new deep-ocean project together with the deliverables of another project helps to generate and maintain momentum, and/or create accountability over the long term. If the project falls within the bounds of a stated goal of a government (e.g. regional and national conservation and sustainable-use goals related to CBD post-2020 Global Biodiversity Framework), NGO, individual or other organization or entity, that person/group becomes a natural partner invested in the project, and can help to create capacity, oversight, support—and eventually, independence—toward these types of projects, in the long term. If this cannot be achieved, then a single project should at least be well-resourced enough to stand alone and be sustained, with a clearly defined start and end date, goals, methods and deliverables determined by the in-country entity to achieve their own priorities and goals.

While the best-laid plans are always subject, and indeed likely, to change, it is nonetheless critical to plan as well in advance as possible. Both pilot studies suffered from technology and equipment timing issues, and/or from the inability to source replacement equipment or parts in-country. Given the unavoidable challenges associated with international travel (and this is especially relevant in COVID-19 times), providing ample time and backup for equipment arrival and repair (if necessary) is critical to project success. Similarly, providing ample time for training and deployment is key, as repeated deployments generate more familiarity and comfort with the technology and equipment, which will likely lead to more use of the technology post-training. More in-field contact time also increases opportunities for stochastic equipment failure and subsequent repair, providing more hands-on opportunities to troubleshoot emerging issues together, again leading to increased familiarity and comfort with the technology.

Finally, though workshops and projects can develop short-term capacity successfully, lasting capacity and independence is better achieved if grounded with experience and/or traditional education, which can also be achieved through long-term and inclusive partnerships [31]. This raises the need for a global push towards appropriate metrics for capacity building [10]. Most deep-ocean scientists have university education and training (including postgraduate), as well as postdoctoral training and in-the-field experience. This level of training and experience cannot be achieved through any workshop or short-term project. Allocating resources for scholarships and experiential long-term training opportunities, including mentorships, is critical to enable lasting capacity that can meet evolving needs and priorities for the country, and keep up with new and emerging technologies, hypotheses, ideas and data that are rapidly advancing in deep-ocean exploration and research.

## Data accessibility

This article has no additional data.
